# Correction: Hom et al. Cognitive Function during the Prodromal Stage of Alzheimer’s Disease in Down Syndrome: Comparing Models. *Brain* *Sci.* 2021, *11*, 1220

**DOI:** 10.3390/brainsci12070925

**Published:** 2022-07-15

**Authors:** Christy L. Hom, Katharine A. Kirby, Joni Ricks-Oddie, David B. Keator, Sharon J. Krinsky-McHale, Margaret B. Pulsifer, Herminia Diana Rosas, Florence Lai, Nicole Schupf, Ira T. Lott, Wayne Silverman

**Affiliations:** 1 Department of Psychiatry and Human Behavior, University of California, Orange, CA 92868, USA; dbkeator@uci.edu; 2 Center for Statistical Consulting, University of California, Irvine, CA 92697, USA; kathark@uci.edu (K.A.K.); jricksod@uci.edu (J.R.-O.); 3 Institute for Clinical and Translational Sciences, University of California, Irvine, CA 92697, USA; 4 New York State Institute for Basic Research in Developmental Disabilities, Staten Island, NY 10314, USA; sharon.krinsky-mchale@opwdd.ny.gov; 5 Department of Psychiatry, Massachusetts General Hospital, Harvard Medical School, Boston, MA 02114, USA; mpulsifer@mgh.harvard.edu; 6 Department of Neurology, Massachusetts General Hospital, Harvard Medical School, Boston, MA 02114, USA; rosas@helix.mgh.harvard.edu (H.D.R.); flai@partners.org (F.L.); 7 Department of Neurology, College of Physicians and Surgeons, Columbia University, New York, NY 10027, USA; ns24@cumc.columbia.edu; 8 Department of Epidemiology, School of Public Health, Columbia University, New York, NY 10027, USA; 9 Department of Pediatrics, University of California, Orange, CA 92868, USA; itlott@uci.edu (I.T.L.); wsilverm@uci.edu (W.S.)

We would like to submit the following correction to our recently published paper [1].

## Error in Tables

In the original publication, there were mistakes in Table 2 and Table 5 as published. The standard deviation listed for the CS group on the DSMSE language test was mistakenly listed as 0.07 in Table 2. The *p*-value listed for the RADD-2 Expressive Language in Table 5 was mistakenly listed as <.001 in Table 5. The Estimate value for the Purdue Pegboard was mistakenly listed as 0615 in Table 5. The corrected Table 2 and Table 5 appear below.

The authors apologize for any inconvenience caused and state that the scientific conclusions are unaffected. This correction was approved by the Academic Editor. The original publication has also been updated.

## Figures and Tables

**Table 2:** Means and standard deviations of raw test scores by AD status and their hypothesized cognitive domain.

Variable (Range of Scores)	Domain	CS (*n* = 103)	MCI-DS (*n* = 41)	Mann–Whitney U (*p*-Value)
Block Design (0–54)	Visuomotor	23.79 (10.49)	17.37 (12.78)	3.11 (0.002)
Boston Naming (0–27)	Language	15.88 (5.51)	13.23 (6.91)	2.04 (0.041)
Category Fluency (0–17)	EF	8.23 (3.18)	6.82 (3.68)	2.21 (0.027)
Cats and Dogs Switch (--17.00–61.80) ^†^	EF	9.80 (11.22)	5.05 (11.21)	1.82 (0.069)
Cued Recall (3–35)	Memory	28.61 (6.83)	21.38 (9.22)	4.43 (0.0001)
DSMSE Language (3–52)	Language	37.10 (9.07)	30.80 (9.63)	3.28 (0.001)
DSMSE Memory (0–23)	Memory	14.08 (4.68)	9.80 (4.57)	4.62 (0.0001)
DSMSE Visual Spatial (2–8)	Visuomotor	6.18 (1.09)	5.59 (1.01)	2.98 (0.003)
mMMSE-DS Anomia (4–20)	Language	18.18 (2.20)	16.49 (4.19)	1.55 (0.122)
mMMSE-DS Concentration (0–6)	EF	3.69 (2.10)	2.39 (2.14)	3.15 (0.002)
mMMSE-DS Fine Motor (1–10)	Visuomotor	8.04 (1.29)	7.05 (2.28)	2.58 (0.010)
mMMSE-DS Orientation (5–30)	EF	25.46 (5.33)	20.87 (6.41)	4.75 (0.0001)
Purdue Pegboard Both Hands (0–8)	Visuomotor	2.62 (1.82)	1.51 (1.71)	3.14 (0.002)
RADD-2 Digit Span Forward (0–8)	EF	4.02 (1.66)	2.97 (1.80)	2.84 (0.005)
RADD-2 Expressive Lang. (0–16)	Language	11.24 (3.92)	8.80 (4.01)	3.34 (0.001)
RADD-2 Receptive Lang. (0–12)	Language	7.56 (2.51)	6.80 (2.99)	1.08 (0.279)
RADD-2 Sensorimotor (0–7)	Visuomotor	6.70 (0.50)	5.85 (1.50)	3.87 (0.0001)
RADD-2 Similarities (0–4)	Language	2.51 (1.49)	1.56 (1.47)	3.40 (0.001)
Rivermead Recognition (0–10)	Memory	5.42 (3.77)	2.21 (3.40)	3.90 (0.0001)
Selective Reminding Test (1–24)	Memory	15.50 (5.62)	9.18 (4.32)	5.70 (0.0001)
Tinetti Gait (4–12)	Visuomotor	10.75 (1.62)	10.37 (1.88)	1.47 (0.143)
VMI (1–25)	Visuomotor	15.45 (3.22)	13.98 (3.40)	1.75 (0.081)

^†^ Cats and Dogs is measured in seconds; all other scores are measured in points (number correct). EF = executive function.

**Table 5:** The relationship between sex, premorbid ID, MCI-DS, and cognitive functioning.

Cognitive Domain	Predictor Variable	Estimate	SE	CR	Standardized Regression Weight	*p*-Value
Structural						
Language/EF	Sex	−0.010	0.075	−0.13	−0.01	0.893
Language/EF	PID	−0.413	0.067	−6.15	−0.41	<0.001
Language/EF	MCI-DS	−0.238	0.074	−3.23	−0.24	0.001
Memory	Sex	0.008	0.076	0.11	0.01	0.916
Memory	PID	−0.241	0.075	−3.21	−0.24	0.001
Memory	MCI-DS	−0.471	0.67	−7.07	−0.47	<0.001
Visuomotor	Sex	−0.093	0.081	−1.14	−0.09	0.255
Visuomotor	PID	−0.302	0.077	−3.91	−0.30	<0.001
Visuomotor	MCI-DS	−0.303	0.78	−3.90	−0.30	<0.001
Measurement						
Language/EF	Boston Naming	0.879	0.023	38.18	0.88	<0.001
Language/EF	DSMSE Language	0.878	0.023	37.81	0.88	<0.001
Language/EF	mMMSE Anomia	0.742	0.041	18.14	0.74	<0.001
Language/EF	mMMSE Concentration	0.724	0.043	16.53	0.72	<0.001
Language/EF	RADD-2 Digit Span Forward	0.782	0.036	21.93	0.78	<0.001
Language/EF	RADD-2 Expressive Language	0.874	0.023	37.52	0.87	<0.001
Language/EF	RADD-2 Receptive Language	0.683	0.047	16.53	0.68	<0.001
Language/EF	RADD-2 Similarities	0.760	0.038	20.05	0.76	<0.001
Memory	Cued Recall	0.673	0.053	12.65	0.67	<0.001
Memory	DSMSE Memory	0.858	0.032	27.09	0.86	<0.001
Memory	Rivermead Recognition	0.702	0.051	13.87	0.70	<0.001
Memory	Selective Reminding Test	0.845	0.033	25.65	0.85	<0.001
Visuomotor	Block Design	0.849	0.033	26.09	0.85	<0.001
Visuomotor	DSMSE Visual Spatial	0.797	0.038	17.05	0.80	<0.001
Visuomotor	Purdue Pegboard	0.615	0.59	10.39	0.62	<0.001
Visuomotor	RADD Sensorimotor	0.624	0.058	10.70	0.62	<0.001
Visuomotor	VMI	0.708	0.048	14.77	0.71	<0.001

CR = critical ratio, EF = executive function, MCI = mild cognitive impairment, PID = premorbid level of ID, SE = standard error.
