# Supplementary material for: Cognitive Function during the Prodromal Stage of Alzheimer’s Disease in Down Syndrome: Comparing Models
Source: Brain Sci. 2021 Sep 16;11(9):1220. doi: 10.3390/brainsci11091220 (PMC8471085; doi:10.3390/brainsci11091220)
Supplement: Supplementary file 1 [file brainsci-11-01220-s001.zip › brainsci-1317473-supplementary.pdf]

Supplementary Figure S1. Effects of aging on cognitive tests by Alzheimer's disease status

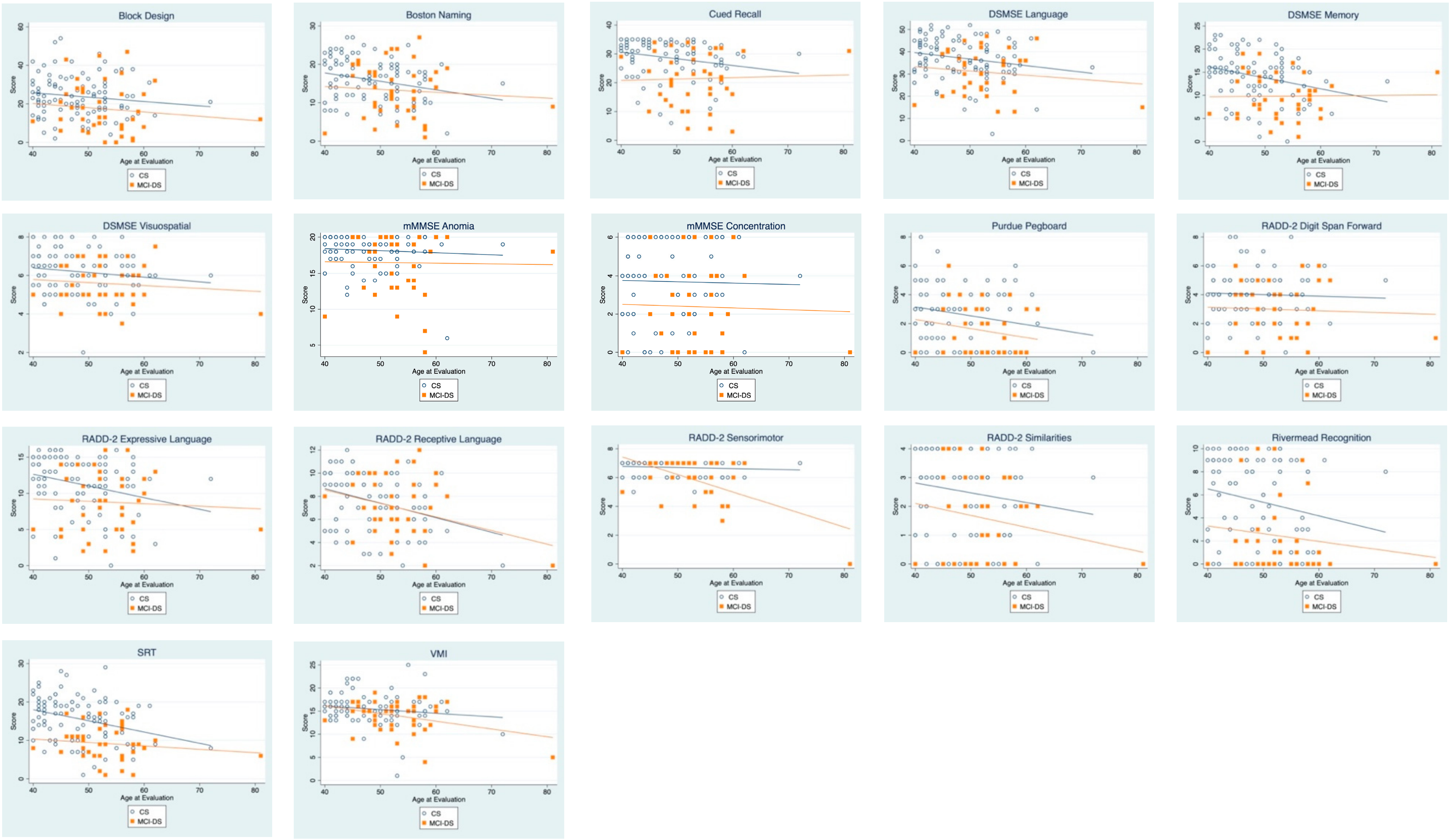

DSMSE = Down Syndrome Mental Status Examination, mMMSE = Modified Mini Mental Status Examination, RADD-2 = Rapid Assessment of Developmental Disabilities, 2nd Edition, SRT = Selective Reminding Test, VMI = Beery Developmental Test of Visual Motor Integration

Aging was associated with lower performance on 9 of the cognitive tests in our battery, controlling for disease status. Disease status did not accelerate age-related decline except for on the RADD-2 Sensorimotor scale.
